# Efficacy and safety of Buyang Huanwu-Tang (Boyang Hwano-Tang) in patients with vascular dementia: A protocol of systematic review and meta analysis

**DOI:** 10.1097/MD.0000000000025886

**Published:** 2021-05-28

**Authors:** Hye Jeong Kook, Da Woon Kim, Ju Yeon Kim, Sang Ho Kim, In Chul Jung

**Affiliations:** a College of Korean Medicine, Daejeon University, Daejeon, Republic of Korea; b Department of Neuropsychiatry of Korean Medicine, Pohang Korean Medicine Hospital, Daegu Haany University, Pohang; c Department of Oriental Neuropsychiatry, College of Korean Medicine, Daejeon University, Daejeon, Republic of Korea.

**Keywords:** Buyang Huanwu-Tang (Boyang Hwano-Tang), dementia, protocol, systematic review, vascular dementia

## Abstract

**Background:**

Vascular dementia (VaD) is the second most common type of dementia; it has a significant impact on patients and exerts a great social and economic burden. However, there has been no comprehensive systematic review assessing the efficacy and safety of Buyang Huanwu-Tang (Boyang Hwano-Tang, BHT) for VaD. Therefore, this protocol was developed to conduct a comprehensive systematic review and meta-analysis to evaluate the effectiveness and safety of BHT in the treatment of VaD.

**Methods:**

We will perform a comprehensive electronic search including MEDLINE, EMBASE, Cochrane Central Register of Controlled Trials, Allied and Complementary Medicine Database, Cumulative Index to Nursing and Allied Health Literature, PsycARTICLES, Oriental Medicine Advanced Searching Integrated System, Korean Studies Information Service System, Research Information Service System, Korean Medical Database, KMbase, National Digital Science Library, China National Knowledge Infrastructure, Wanfang database, VIP database, Citation Information by NII, and other sources from their inception to November 25, 2020. This systematic review will include only randomized controlled clinical trials of BHT for VaD. The main outcomes will include the Mini-Mental State Examination, Montreal Cognitive Assessment, and Revised Hasegawa's Dementia Scale. Two researchers will independently conduct study selection, data extraction, and appraise the quality and risk of bias of the included studies. A meta-analysis will be conducted using Review Manager version 5.4. The evidence quality of each outcome will be appraised according to the Grades of Recommendation, Assessment, Development, and Evaluation.

**Results:**

This study will provide comprehensive understanding of the efficacy and safety of BHT for the treatment of VaD.

**Conclusions:**

The findings of this study will provide reliable evidence for clinical application and further study of BHT for VaD.

**Ethics and dissemination:**

Ethical approval is not required because individual patient data will not be included in this study. The study findings will be disseminated through conference presentations.

**OSF registration DOI:**

10.17605/OSF.IO/NDYGP

## 1 Introduction

Dementia is widely known for causing memory loss and cognitive impairment, which usually develops in the elderly.^[1]^ As life expectancy increases, dementia also rapidly grows as a global public health problem with nearly 10 million new cases worldwide annually; it is expected to reach 82 million cases in 2030 and 152 million in 2050 according to WHO.^[2]^ Dementia burdens governments, communities, families, and individuals with increasing costs and losses in productivity for economies.^[2]^ This burden shows an annual increase between 2000 and 2016. The total economic costs caused by dementia increased with an annual growth rate of 15.94%, which included the cost of informal care with an annual growth rate of 21.50%.^[3]^

Vascular dementia (VaD) is widely recognized as the second most common type of dementia after Alzheimer's disease (AD).^[4]^ In a recent meta-analysis of multiple treatments for cognitive dysfunction in dementia, symptomatic treatment for VaD was the most effective intervention among various treatments available for dementia.^[5]^ Recently, cholinesterase inhibitors and memantine have been widely used for treatment; however, they show significantly increased risks for adverse events, including anorexia, nausea, vomiting, and diarrhea.^[6]^ According to a meta-analysis, compared to the placebo-treated control group, the cholinesterase inhibitor-treated group showed significant improvement in cognitive function score but showed a two-fold increase in the odds of discontinuation due to adverse events.^[7]^ Additionally, in a recent systematic review and meta-analysis, using cholinesterase inhibitors or memantine for patients diagnosed with AD or VaD showed modest treatment effects on cognitive function compared to the control group, which was monitored via Mini-Mental State Examination (MMSE).^[8]^ Thus, the efficacy and safety of conventional Western treatments have been questioned, and the need for alternative treatments has been highlighted. Several alternative treatments, including acupuncture and herbal medicine, have been suggested.^[9–11]^ A meta-analysis of randomized control trials (RCTs) showed that the use of herbal medicine for VaD could improve cognitive function as well as activities of daily living, have comparable clinical effectivity rates compared to Western conventional treatments, are more beneficial in improving MMSE when compared to placebo, and have fewer adverse events than that of the placebo or Western conventional treatment group.^[10]^ In addition, original or modified BHT was the most widely used herbal medicine that significantly improved neurologic deficits and activities of daily living when used in combination with conventional treatment in patients after ischemic stroke.^[11]^ In addition, acupuncture, when combined with Buyang Huanwu Tang (Boyang Hwano Tang, BHT), reduced the recurrence rate of ischemic stroke compared to acupuncture alone.^[12]^ Therefore, we present the protocol for a systematic review and meta-analysis to investigate the use of BHT in treating cognitive dysfunction in patients with VaD.

BHT is a herbal medicine traditionally known to treat hemiplegia, facial nerve palsy, difficulty in speaking, drooling, dry stool, frequent urination, and fecal incontinence. It was first used in Yilin Gaicuo (Correction on Errors in Medical Classics).^[13]^ It is a well-known herbal medicine used for treating hemiplegia caused by ischemic stroke due to Qi deficiency and blood stasis syndrome.^[14]^ There are studies showing that BHT improves neurological deficits with substantial neuroprotective effects in both animal models and humans,^[15,16]^ and seems generally safe in patients with acute ischemic stroke.^[17]^ BHT upregulated cellular signaling proteins related to neural stem cell proliferation and differentiation, neuronal and astrocytic differentiation, and axon formation; as well as showed anti-apoptotic effects.^[18]^ In addition, BHT activates neuroprotective signaling cascades,^[18]^ protects Schwann cells from oxidative injury,^[19]^ and activates neuro-generation signaling cascades.^[18]^ BHT can also activate the immune system and exert anti-inflammatory effects.^[19,20]^ The angiogenetic effect of BHT after cerebral ischemia is known to increase vascular endothelial growth factor-related pathways, such as angiopoietin expression in the hippocampus.^[19,21]^

There was a recent meta-analysis on the effect and safety of BHT combined with Western medicine for VaD treatment, but it was not comprehensive and had poor report quality and data analysis.^[22]^ In addition, a systematic review on the mechanism of BHT made use of only preclinical studies.^[19]^ There was also a meta-analysis of BHT in a sequela of apoplexy, but the intervention was combined with acupuncture and not utilized as monotherapy.^[23]^ Therefore, we aim to carry out a more comprehensive and normative systematic review and meta-analysis to provide objective and scientific evidence on the use of BHT as a treatment for VaD. We hope to garner clear evidence regarding the use of BHT in patients with VaD, ultimately leading to significant improvement of VaD patients’ cognitive impairment along with lowering their socioeconomic burden with BHT treatment.

## 2 Materials and methods

### 2.1 Study registration

This systematic review protocol has been registered on the Open Science Framework (OSF) with a registration DOI of 10.17605/OSF.IO/NDYGP. The protocol was strictly developed under the guidelines of the Preferred Reporting Items for Systematic Reviews and Meta-analyses Protocols (PRISMA-P).^[24]^

### 2.2 Inclusion and exclusion criteria

#### 2.2.1 Types of included studies

We will only include RCTs in this systematic review investigating the effects of BHT in patients with VaD. In addition, studies using parallel and crossover designs will be included, whereas other studies, such as case reports, case series, literature reviews, and uncontrolled trials, will be excluded. There will be no language restrictions during the database search, but all search words will be in English, Chinese, or Korean.

#### 2.2.2 Types of participants

Participants with major neurocognitive disorders and diagnosed with VaD according to certain diagnostic criteria will be included. All patients should be diagnosed with VaD according to at least one of the following criteria: the Diagnostic and Statistical Manual of Mental Disorders or International Classification of Diseases criteria, the Chinese Classification of Mental Disorders criteria, the Alzheimer's Disease Diagnosis and Treatment Center criteria, or the National Institutional of Neurological Disorders and Stroke and Association Internationale pour la Recherche et l’Enseignement en Neuroscience criteria. There will be no restrictions on age, sex, nation, race, inpatient or outpatient status, or medical history. Studies of patients with major neurocognitive disorders other than VaD or other diseases, healthy elderly people with delirium, or with normal cognitive degradation will be excluded.

#### 2.2.3 Types of intervention

##### 2.2.3.1 Experimental intervention

Studies using BHT alone or BHT combined with anti-dementia drugs as interventions will be included. Routine care for stroke, such as anti-hypertensives and antithrombotic drugs, will be allowed regardless of whether they were combined. BHT in various formulas, such as decoctions, capsules, granules, and powder, will be included. Modified prescriptions will be included for as long as they include the basic composition of BHT. There will be no restrictions on age, sex, nation, race, inpatient or outpatient status, or medical history.

##### 2.2.3.2 Control intervention

Control groups will be those taking placebo or receiving routine care for VaD. Those taking anti-dementia drugs or those on the waiting-list control group will be included. Studies that used herbal medicines as controls will be excluded.

#### 2.2.4 Types of outcome measures

##### 2.2.4.1 Primary outcomes

The primary outcomes will be scores on the MMSE, Montreal Cognitive Assessment, Hasegawa's Dementia Scale (HDS) and Revised Hasegawa's Dementia Scale (HDS-R).

##### 2.2.4.2 Secondary outcome

The secondary outcomes will be as follows:

1. Activities of Daily Living assessed using the Barthel Index and Modified Barthel Index will be selected for daily living function.
2. The effective rate based only on the MMSE and HDS will be included.
3. Adverse events and medical examinations for safety evaluation will be recorded.

### 2.3 Search methods

Two independent researchers (DW Kim and HJ Kook) will search the following electronic bibliographic databases from their inception dates to November 25, 2020: MEDLINE via PubMed, EMBASE via Elsevier, the Cochrane Central Register of Controlled Trials, Allied and Complementary Medicine Database via EBSCO, Cumulative Index to Nursing and Allied Health Literature via EBSCO, PsycARTICLES via ProQuest, Oriental Medicine Advanced Searching Integrated System, Korean Studies Information Service System, Research Information Service System, Korean Medical Database, National Digital Science Library via KISTI, China National Knowledge Infrastructure, Wanfang Database, VIP Database, and Citation Information by NII. We will also search the reference lists of the relevant articles and manually search for additional gray literature using Google Scholar for inclusion. There will be no language restrictions.

### 2.4 Data collection and analysis

#### 2.4.1 Literature selection.

We will use the EndNote X9.3.3 software (Clarivate Analytics, Boston, USA) to record and organize the electronic literature retrieved from the databases mentioned. The study selection will be conducted by two reviewers (HJ Kook, DW Kim) by independently assessing titles and abstracts after removing duplicates from all search results. Full texts will then be obtained and assessed for inclusion and exclusion criteria. If no consensus is reached, we will try to resolve disagreements on the eligibility of studies through discussion or by asking the third experienced review authors (SH Kim, IC Jung). Two independent reviewers will extract the data of interest from the eligible studies and fill in the data collection sheet. Any disagreement will be resolved by consensus or consultation with the two experienced review authors (SH Kim, IC Jung).

#### 2.4.2 Data extraction

Two review authors (HJ Kook, DW Kim) will independently read and extract data from selected studies. If no consensus is reached, we will try to resolve disagreements through discussion or by asking the two experienced review authors (SH Kim, IC Jung). We will use a standardized data extraction form that includes the source, first and corresponding author, year of publication, study design, characteristics of participants including diagnostic criteria, intervention, comparator, duration, follow-up, outcome measurement, results, and adverse events according to the Cochrane Handbook for Systematic Reviews of Interventions (version 6.1.0) (Chapter 5.3).^[25]^ Microsoft Excel 2016 will be used for data and information management.

### 2.5 Assessment of bias risk and quality of included studies

Two researchers (DW Kim, HJ Kook) will independently assess the risk of bias in the selected RCTs. The risk of bias tool presented in the Cochrane Handbook for Systematic Reviews of Interventions (version 6.1.0) will be used to assess the quality of the studies included in this review in Review Manager (RevMan, version 5.4, The Cochrane Collaboration, London, UK). The following characteristics will be considered: random sequence generation, allocation concealment, blinding, incomplete outcome data, selective reporting, and other biases. Each potential trial of bias will be graded as high, low, or unclear. When two quality reviewers are unable to reach a consensus through negotiation on risk assessment, the two experienced review authors (SH Kim, IC Jung) will make the decision.

### 2.6 Data analysis

#### 2.6.1 Measures of treatment effect

Where studies have used the same type of interventions and comparators with the same outcome measures, statistical analyses will be performed using Review Manager (RevMan, version 5.4). Mean differences (MDs) or standard mean differences (SMD) with 95% confidence intervals (CIs) will be used as continuous data. For the analysis of dichotomous outcomes, data risk ratios (RRs) with 95% CIs were calculated.

#### 2.6.2 Dealing with missing data

Missing data and data errors will be thoroughly reported. For every included study, missing data or incomplete information, including the quantities of withdrawals and exclusions, will be gathered and requested by the corresponding author via email or telephone. If we fail to obtain the related data, we will assume missing outcomes for participants not obtaining any improvement in their clinical outcome variables. For continuous missing outcomes, we will attempt to calculate mean deviation and standard deviation values as the first option when the medians, *P* values, or CIs are recorded in the included studies. When necessary, the possible impact of missing data on the final findings of the review will be disclosed in the discussion section. If the methodology does not provide a detailed explanation, the corresponding study's risk of bias will be judged as unclear.

#### 2.6.3 Assessment of heterogeneity

Heterogeneity between the studies in terms of effect measures will be assessed using both the χ^2^ test and the I^2^ statistic, and we will consider an I^2^ value greater than 50% as indicative of substantial heterogeneity and a value greater than 75% as indicative of serious heterogeneity.

#### 2.6.4 Assessment of reporting biases

If sufficient studies are available, we will assess evidence of publication bias using a funnel plot. The results will be pooled using a random-effects model if the included studies have significant heterogeneity, while a fixed-effect model will be used if the heterogeneity is not significant or if the number of studies included in the meta-analysis is very small, meaning that the estimate of the between-study variance will lack precision.

#### 2.6.5 Data synthesis

We will provide a narrative synthesis of the findings from the included studies. For example, the demographic characteristics of the participants as well as details of the experimental and control interventions, outcomes, and results, will be provided. For data errors, after being gathered, we will try to contact the corresponding author via email or telephone for correct data, but if there is no response, we will exclude the data from the data synthesis.

Where studies have used the same type of interventions and comparators with the same outcome measures, we will pool the results using the Review Manager software (version 5.4; The Cochrane Collaboration, London, UK), with MDs or SMDs for continuous outcomes and RRs for binary outcomes, and 95% CIs.

#### 2.6.6 Subgroup analysis

If the necessary data are available, we will conduct a subgroup analysis according to the duration of treatment and the type of Western medicine used in the intervention.

#### 2.6.7 Summary of evidence

Two researchers (DW Kim and HJ Kook) will independently assess the quality of evidence. If no consensus is reached, we will try to resolve disagreements regarding the eligibility of studies through discussion or asking the two experienced review authors (SH Kim, IC Jung). We will use the Grades of Recommendation, Assessment, Development, and Evaluation to assess the quality of evidence.^[26]^ For the assessment scale, the confidence in each outcome will be divided into four levels: high, medium, low, and extremely low.

## 3 Discussion

Herbal medicine has a long history and is widely used for various diseases, including dementia. BHT is a herbal medicine that is widely used to treat stroke as well as the sequelae of cerebral hemorrhage, cerebral thrombosis, coronary artery disease, and VaD. In Korean medicine, BHT is used to treat cerebrovascular diseases, including stroke or VaD, through its ability to invigorate the circulation by tonifying Qi. Preclinical and clinical studies have shown that original or modified BHT increases cerebral blood flow^[27–29]^ and has neuroprotective^[18,19,21,30–32]^ as well as anti-inflammatory^[15,19,33]^ effects.

Following this protocol, we will conduct a systematic review and meta-analysis of RCTs of BHT combined with or without routine care or anti-dementia drugs in the treatment of VaD. We will strictly evaluate the quality of the included trials. This systematic review will summarize the current state of BHT use for VaD. Based on the results of previous studies, we will evaluate the efficacy and safety of BHT through quality assessment of up-to-date studies, which may give insight on how to conduct further clinical research on the subject.

## Acknowledgments

We would like to thank Editage (www.editage.co.kr) for English language editing.

## Author contributions

**Conceptualization:** Ju Yeon Kim, Sang Ho Kim, In Chul Jung.

**Data curation:** Hye Jeong Kook, Da Woon Kim.

**Formal analysis:** Hye Jeong Kook, Da Woon Kim.

**Funding acquisition:** In Chul Jung.

**Writing – original draft:** Hye Jeong Kook, Da Woon Kim.

**Writing – review & editing:** Hye Jeong Kook, Ju Yeon Kim, Sang Ho Kim, In Chul Jung.
